# Dual Linkage of a Locus to Left Ventricular Mass and a Cardiac Gene Co-Expression Network Driven by a Chromosome Domain

**DOI:** 10.3389/fcvm.2014.00011

**Published:** 2014-12-10

**Authors:** Marie-Pier Scott-Boyer, Samantha D. Praktiknjo, Bastien Llamas, Sylvie Picard, Christian F. Deschepper

**Affiliations:** ^1^Cardiovascular Biology Research Unit, Institut de recherches cliniques de Montréal (IRCM), Université de Montréal, Montréal, QC, Canada

**Keywords:** cardiac complex traits, genetics of gene expression, weighted gene co-expression network analysis, mouse recombinant inbred strains, chromosome domain, cardiac left ventricular mass

## Abstract

We have previously reported *Lvm1* as a quantitative trait locus (QTL) on chromosome 13 that links to cardiac left ventricular mass (LVM) in a panel of AxB/BxA mouse recombinant inbred strains (RIS). When performing a gene expression QTL (eQTL) analysis, we detected 33 cis-eQTLs that correlated with LVM. Among the latter, a group of eight cis-eQTLs clustered in a genomic region smaller than 6 Mb and surrounding the *Lvm1* peak on chr13. Co-variant analysis indicated that all eight genes correlated with the phenotype in a causal rather than a reactive fashion, a finding that (despite its functional interest) did not provide grounds to prioritize any of these candidate genes. As a complementary approach, we performed weighted gene co-expression network analysis, which allowed us to detect 49 modules of highly connected genes. The module that correlated best with LVM: (1) showed linkage to a module QTL whose boundaries matched closely those of the phenotypic *Lvm1* QTL on chr13; (2) harbored a disproportionately high proportion of genes originating from a small genomic region on chromosome 13 (including the 8 previously detected cis-eQTL genes); (3) contained genes that, beyond their individual level of expression, correlated with LVM as a function of their inter-connectivity; and (4) showed increased abundance of polymorphic insertion–deletion elements in the same region. Taken together, these data suggest that a domain on chromosome 13 constitutes the biologic principle responsible for the organization and linkage of the gene co-expression module, and indicate a mechanism whereby genetic variants within chromosome domains may associate to phenotypic changes via coordinate changes in the expression of several genes. One other possible implication of these findings is that candidate genes to consider as contributors to a particular phenotype should extend further than those that are closest to the QTL peak.

## Introduction

The identification of gene variants causally linked to complex phenotypic traits still remains difficult. Functional genomic studies, which evaluate the functional consequences of genetic variations on intermediate molecular traits, have been proposed as a means to improve the power of detecting such gene variants (1, 2). The importance of gene expression for complex traits is illustrated by studies showing that trait-associated polymorphisms are more likely to also be associated with expression of particular genes (3), and that variants associated with common human diseases involve predominantly regulatory DNA sequences (rather than protein-coding regions) (4). Accordingly, gene expression constitutes one type of intermediate molecular phenotypes that has been studied often, with quantitative trait loci (QTLs) linked to gene expression being called “expression QTLs (eQTLs)” (2). When the expression of a given gene associates with a genetic polymorphism that maps close to that gene’s locus, the corresponding eQTL is referred to as a “cis-eQTL,” with the presumption that a cis-acting polymorphism within the regulatory machinery of that gene affects its expression. *Cis-eQTLs* that both *colocalize* with the phenotypic QTL and correspond to genes whose expression *correlates* with quantitative variation of the phenotype have been called “c3-eQTLs,” and have been used to prioritize genes to be considered as candidates harboring causal mutations (5). However, there are several limitations to this strategy: (1) dysregulation of single genes is believed to account for only a minority of complex quantitative traits (6), while epistatic interactions may represent important components of the architecture of complex traits (7); (2) the abundance of eQTLs and the strong correlation structure in the genome is such that some of their overlaps with phenotypic QTLs may often be coincidental and not driven by the same functional variants (8); and (3) instead of representing the sum of the individual actions of several independent biomolecules, biological systems are more typically organized as modular networks (9, 10).

Since functionally related genes are likely to show mutual dependence in their expression network, one alternative to the identification of c3-eQTLs has been to construct gene co-expression networks, with the aim of defining highly inter-connected gene modules and identify which ones correlate best with variations in complex traits (9–11). One underlying assumption of this strategy is that it may be easier to predict (on the basis of concordant gene annotations) the function of a module rather than that of individual genes (10, 11). Accordingly, it has been possible in some cases to find within modules enrichment for genes originating from particular biologic pathways (11–14). However, such genes usually represent only a small fraction of genes in the module, and their identification is not sufficient to identify how genetic determinants may lead to coordinate changes in the expression of all genes in the module. Alternatively, genetic mapping of “eigengenes” (which represent the first principal component of all expression profiles in modules) has shown that entire modules could be linked to QTLs and that some of such “module-QTLs (mQTLs)” may have profiles matching that of phenotypic QTLs (15, 16). Although such findings suggest that the same genetic determinants may link to both a phenotype and the expression levels of genes within the associated module, the nature of such variants remains to be elucidated.

Interestingly, by analyzing datasets of gene expression in several tissues from mouse recombinant inbred strains (RIS), we found recently that close to 30% of the gene co-expression modules detected in such datasets showed genetic linkage to a mQTL (17). For the majority of such modules, the mQTL was on the same chromosome as the one contributing most genes to the module, with genes originating from that chromosome showing higher connectivity than other genes in the module. Along with the fact that corresponding genomic regions contained increased abundance of polymorphic structural variants, these data suggested that such modules were driven by particular chromosome domains (17, 18). Beyond individual c3-eQTLs, it is thus possible that such chromosome domain-driven (CDD) mQTLs link to a quantitative phenotype via coordinate changes in the expression of several genes in proximity to the mQTL.

Using a panel of 24 AxB/BxA mouse RIS, we have also previously shown that chromosome 13 (chr13) harbors one major QTL linked to cardiac left ventricular mass (LVM) (identified as “*Lvm1*”) (19). LVM is a highly heritable quantitative complex trait that constitutes an important and independent predictor of cardiovascular mortality and morbidity (20, 21). To further test whether genetic variants could link to changes in LVM via coordinate changes in the expression of several genes, we used gene expression data from the cardiac LVs of four male mice from all 24 strains. We analyzed the data to establish an inventory of all c3-eQTLs and to detect modules of inter-connected genes by weighted gene co-expression network analysis (WGNCA). Within the gene co-expression network, we found that 40% of detected modules showed linkage to an mQTL. The module correlating best with LVM had the characteristics of a CDD module, and contained genes that correlated with the phenotype not just by their individual expression level, but mostly as a function of their inter-connectivity. These findings indicate a mechanism whereby genetic variants may lead to phenotypic modifications via coordinate changes in the expression of several genes within particular chromosome domains.

## Materials and Methods

### Gene expression and mapping analyses

The AxB/BxA mouse RIS originate from reciprocal crosses between the two parental C57BL/6J and A/J inbred strains (22). We have previously used a set of 24 strains from that panel to detect QTLs linked to normalized cardiac LVM (defined as LV weight corrected for whole body weight, and simply referred hereafter as “LVM”) (19). Using four adult male individuals from each of the same strains, we extracted total RNA from the cardiac left ventricles of mice, and used them to profile gene expression using Illumina MouseRef-8 v2.0 BeadChip. All 96 samples were randomized across all lanes in a total of 12 microarray slides, as described previously (23), and hybridized in two separate batches. To avoid the possibility that polymorphisms within probes used for the microarray could affect the gene expression results, we used the Sanger website to verify whether the Illumina probes corresponded to regions containing high-quality SNP polymorphisms (score > 100 according to the Sanger website): only 91 SNPs were detected within the probe sequences, and the corresponding probes were removed for the purpose of gene expression analysis. Possible batch effects were normalized using the ComBat software (24). All processed data are available for public access at GeneNetwork (accession number GN421)^1^. Further analyses, involving genotyping of all 24 RIS and mapping of eQTLs, were performed as described previously (18). All files with genotypes are available at the following Website: https://github.com/raphg/iBMQ/blob/master/data_application_note/data_application_note.R. Gene expression data (corresponding for each strain to the average of values obtained in four individuals per strain) were analyzed (along with genomic maps) with the “QTL” R package (25), using a detection threshold corresponding to a “logarithm-of-the-odds (LOD)” score of 3.3 (26). For each eQTL, we determined whether the transcription was regulated *in cis* or *in trans* by defining cis-eQTLs as those whose peak eQTL was within 1 Mb of the physical location of the corresponding gene start. Confidence intervals were determined by calculating the 1.5-LOD support interval (27). For of each cis-eQTL, we calculated the Pearsons correlation coefficient of the expression level of its corresponding gene with the value of LVM in corresponding strains. To determine which cis-eQTL genes had expression values that correlated significantly with LVM, Westfall–Young adjusted *p*-values were calculated on the basis of 1,000,000 permutations, using R. To find a threshold corresponding to a “false discovery rate (FDR)” = 0.1, adjusted *p*-values were then transformed into *q*-values, using the “*q*-value” R package.

C3-eQTLs represent situations where a cis-eQTL and a phenotypic trait share linkage with a common QTL. Such cases represent “triads” where statistical procedures based on the variance of the traits can be applied to infer causality. In essence, the procedure consists of running reciprocal QTL scans, i.e., scanning for either the phenotypic traits with gene expression levels as covariates, or for the gene expression traits using the phenotypic trait as a covariate (14, 28, 29). The first test scans for cis-eQTLs that (when expression levels of corresponding genes are used as covariates) cause the largest drop in LOD score for the phenotypic QTL. The larger the drop, the more likely it is that gene expression is causal to the phenotype. Conversely, the second test analyzes whether using the phenotype as a covariate causes such a drop in LOD score of a cis-eQTL that it lowers its peak below the significance levels. In such cases, cis-eQTLs are likely to be reactive (instead of causal) to the phenotype (28).

### Gene co-expression networks and modules

On the basis of the expression data of all 8725 genes detected with the Illumina microarray in the LVs of male individuals from all 24 strains, we used the WGCNA R package (30) to construct a gene co-expression network. Network analyses were performed on the basis of the following calculations: (1) estimation of a particular β power value was performed by using the scale-free topology criterion described previously (31), which led us to the power β = 6 value for all groups; (2) measures of topological overlap between nodes were calculated on the basis of the number of shared neighbors; and (3) a hierarchical clustering of the above values was performed to produce dendrograms. Within a network, each gene represents a node, and the connections between nodes are defined as edges. To define modules (i.e., clusters of highly inter-connected genes), branches of the hierarchical clustering tree were cut using the dynamic tree cut algorithm implemented in the dynamicTreeCut R package.

Since WGCNA is a network-based method that requires fine-tuning of several parameters, we have tested the robustness of the inferred co-expression modules to ensure the stability of the results. To test the robustness of our network, we varied the values of four parameters used within network construction and module identification: (1) the soft thresholding β value; (2) the deepSplit and minClusterSize variables of the cutreeDynamic function; and (3) the cutHeight variable (corresponding to the maximum dissimilarity that qualifies modules for merging) of the mergeCloseModules function. To compare our network (with default parameters) to those obtained with varying parameters, we used the ps.cluster function of the genefu R package (available at www.bioconductor.org) to calculate prediction strength values (32) and reported the median prediction strength values of all the clusters. We have also tested the robustness of inferred co-expression modules by looking at the top 20 and 40% most connected genes of each module and testing to which extent these genes were retained in modules when varying the soft thresholding value. We reported the mean percentage of all clusters for the different soft thresholding β values.

“Eigengene” values (defined as the first principal component of module-specific expression data) were then calculated for each module. Since eigengenes can be considered as representatives of the gene expression profiles in corresponding modules, eigengene values can be used to either detect modules correlating with a given phenotype, or to detect “mQTLs,” i.e., QTLs showing linkage to entire gene co-expression modules. Mapping of mQTLs was performed with the “QTL” R package (25), using the same criteria described above. Modules whose eigengene value correlated strongly with LVM (*p* < 0.01) were visualized graphically with the Cytoscape software (33), using the values of connection strength for the edges and that of connectivity (defined as the sum of connection strengths of each node with all other network genes) for each node, as calculated by WGCNA. In some cases, comparisons were performed between several groups of genes within modules, using ANOVA tests followed by Tukey’s *post hoc* multiple comparison tests.

### Analysis of structural variants

A list of mouse genomic structural variants (including deletions, insertions, and copy number variants) was obtained from the Sanger database^2^. Structural variants defined as polymorphic between the parental A/J and C57BL/6J strains were those showing either “insertion” (i.e., present in C57BL/6J but absent in A/J) or “deletion” (i.e., present in A/J but absent in C57BL/6J) vs. the mm9 reference sequence of the whole genome from C57BL/6J. The majority of insertion–deletions in mice are in fact transposable elements, among which short interspersed nuclear elements (SINEs) are the most abundant (34).

A list of SINEs that are polymorphic between the parental A/J and C57BL/6J strains was obtained from a recent publication (35) For certain mQTLs (see below), we examined the abundance of both insertion–deletions (indels) and polymorphic SINEs in consecutive 2 Mb regions extending on both sides of the mQTL peaks (up to total distances of 18 Mb). The profiles of abundance of these elements were compared to those found in regions of similar size surrounding a total of 500 polymorphic SNPs randomly selected in the entire genome. Comparisons between groups were performed by ANOVA followed by Tukey’s *post hoc* multiple comparison tests.

## Results

### Identification of c3-eQTLs in hearts from AxB/BxA mouse RIS

**Figure 1:**
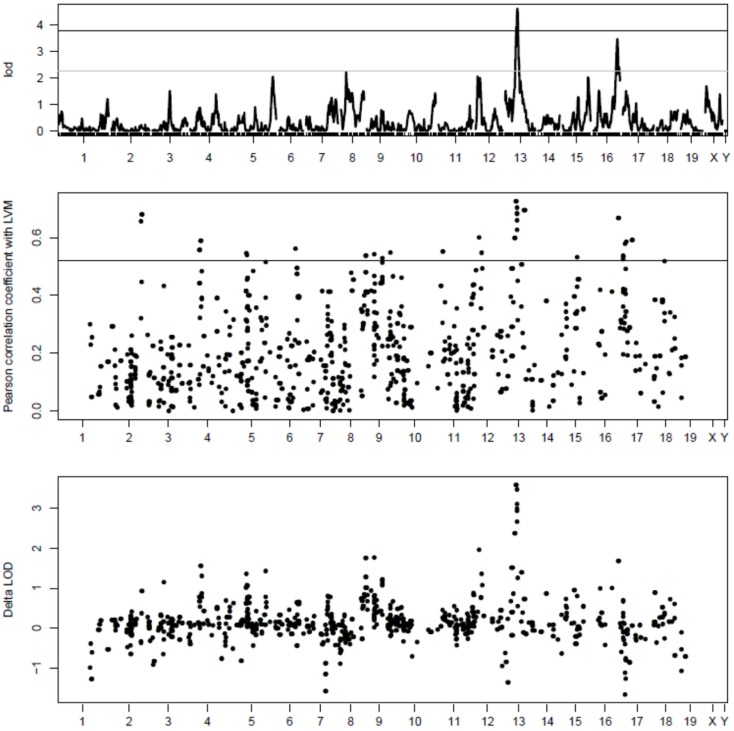
**Top part: QTL mapping analysis of LVM in mouse AxB/BxA RIS**. The number of each chromosome is indicated on the *x* axis; the LOD scores are indicated on the *y* axis; the horizontal plain and dashed lines represent the threshold for significant and suggestive LOD scores, respectively. The strongest QTL is *Lvm1* on chr13. Middle part: c3-eQTL analysis of cardiac cis-eQTLs in mouse AxB/BxA RIS. Similarly as in the top figure, the number of each chromosome is indicated on the *x* axis. Expression levels of 777 cis-eQTLS were correlated with values of normalized LVM; the absolute values of Pearson correlation coefficients are indicated on the *y* axis. The horizontal line represents significance threshold level, as calculated by permutation tests (see Supplementary Material). On chr13, there is a clustering of eight c3-QTLs, each having their peak within the confidence interval of *Lvm1* (see Figures S1A,B in Supplementary Material). On chr17, there is a clustering of five c3-QTLs, each having a profile matching closely that of a QTL showing weak linkage with LVM (see Figure S2 in Supplementary Material). Bottom part: amplitude of the drop in LOD score for *Lvm1* observed when using the expression level of each cis-eQTL gene as a covariate. The largest drops in LOD scores are observed for the eight c3-QTLs on chromosome 13 whose profiles overlap with that of *Lvm1*.

Gene expression profiling with Illumina microarrays allowed us to detect expression of a total of 8725 genes in extracts of cardiac LVs from mouse AxB/BxA RIS and measure the abundance of corresponding mRNAs. Genetic mapping of these gene expression values revealed a total of 10,530 eQTLs above the 3.3 LOD threshold. Among those, 777 loci had a peak that was located within <1 Mb from the transcription start site of the gene whose expression was measured were defined as cis-eQTLs (Figure 1). Out of those, only 33 corresponded to genes whose expression level correlated significantly (FDR < 0.1) with the values of LVM. In this dataset, the threshold corresponded to *r*^2^ values ≥0.54. These 33 cis-eQTLs thus corresponded to c3-QTLs (Figure 1). Strikingly, 8 of 33 c3-eQTLs were clustered between positions 59.74 and 64.53 Mb within an interval of 5.8 Mb on chr13 (i.e., the same chromosome as *Lvm1*) (Table S1 in Supplementary Material). Within that cluster, six cis-eQTL genes corresponded in fact to six contiguous genes all contained within a 250 kb interval. All eight c3-eQTLs had confidence intervals that overlapped with that of *Lvm1* (whose peak was located at position 57.8 Mb on chr13) (Figures S1A,B in Supplementary Material). When scanning the LVM QTL using the expression levels of all cis-eQTL genes as covariates, the same 8 cis-eQTLs on chromosome 13 were the ones that yielded the largest drops in LOD scores for the LVM QTL (Figure 1). When QTL scanning was performed for these eight cis-eQTLs using LVM values as a covariate, residual variance was such that the eQTL peaks were still clearly detectable for all genes (Figure S2 in Supplementary Material).

### Weighted gene co-expression network analyses

On the basis of WGCNA, we detected a total of 49 modules, each containing at least 40 genes and being identified by a color name. We tested the robustness of the network by calculating the prediction strength after varying four different parameters used for network construction and module identification. The results of the network were affected only to a minimal extent by varying the deepSplit and minClusterSize variables (of the cutreeDynamic function) and the cutHeight variable (of the mergeCloseModules function) (Figure S3 in Supplementary Material). Since varying the soft thresholding variable seemed to affect prediction strength to a greater extent (Figure S3 in Supplementary Material), we verified the robustness of inferred co-expression modules by testing how variations of the soft thresholding β value affected the top 20 and 40% most connecting gene of each module. We found that membership of the most connected genes was affected only to a minimal extent by variations of the soft thresholding β value (Figure S4 in Supplementary Material).

**Figure 2:**
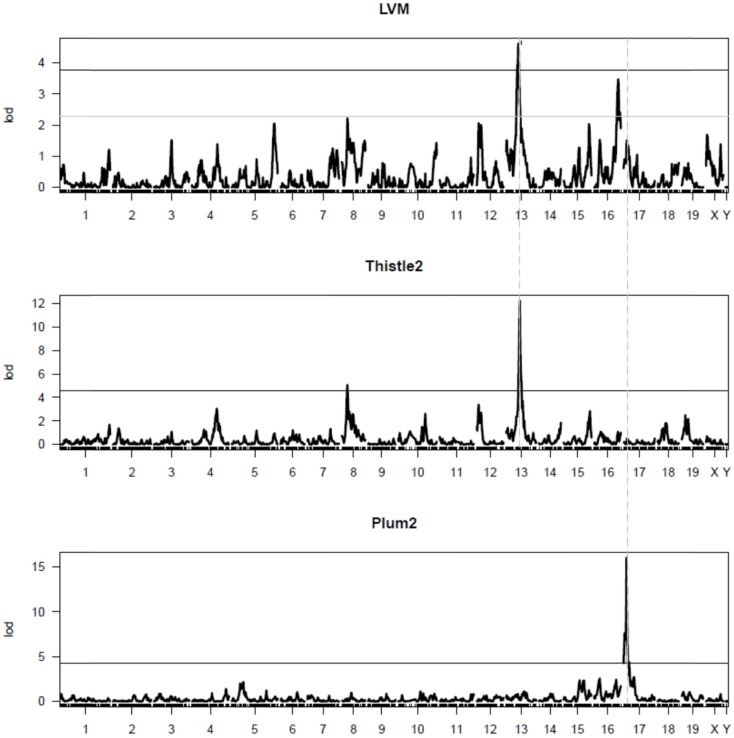
**QTL mapping of the thistle2 and plum2 modules**. The graphs represent the QTL mapping profiles for (1) LVM (top graph), the thistle2 module (middle graph), and the plum2 module (bottom graph), respectively. The major mQTL for thistle2 on chr13 (LOD = 12.2) has a profile matching closely that of *Lvm1* on chr13. The major mQTL for plum2 on chr17 (LOD = 16) had a profile that matched closely that of a minor (and non-significant) LVM QTL on chr17.

Additional analyses were performed using modules identified with the default parameters of WGCNA. By correlating the values of the eigengene of each module with that of the LVM values, two distinct modules were found to correlate significantly (*p* < 0.01) with LVM: (1) the module “thistle2” contained a total of 48 well-annotated genes, and its correlation coefficient with LVM was 0.66 (*p*-value = 0.0004); (2) the module “plum2” contained 49 well-annotated genes, and its correlation coefficient with LVM was -0.57 (*p*-value = 0.004). QTL mapping analyses were performed for the eigengene of these two modules to detect corresponding mQTLs (Figure 2). The mQTL of thistle2 had a strong peak on chr13 (LOD = 12.2) and a profile that matched closely that of *Lvm1*. The mQTL of plum2 module had a strong peak on chr17 (LOD = 16); however, its profile matched only that of a minor (and non-significant) LVM QTL on chr17.

### Properties of co-expression modules correlating with LVM

**Figure 3:**
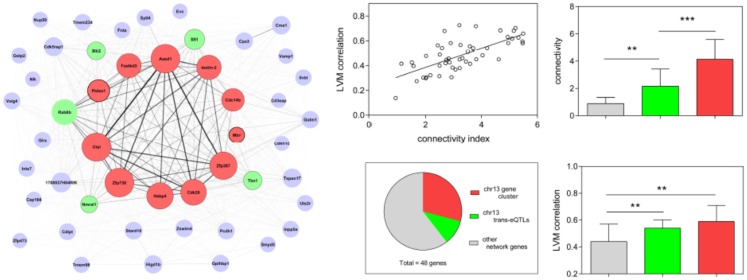
**Diagram representation and properties of the thistle2 co-expression module**. The size of each node is proportional to the connectivity of each corresponding gene; the width of each edge is proportional to the strength of correlation between the two corresponding genes. Each node is color-coded in the following fashion: the red nodes comprised a physical cluster of 11 eQTL genes all contained within a 8 Mb interval on chr 13 (from positions 60.8–68.7 Mb); the green nodes represent 5 trans-eQTL genes, each having a profile also matching that of *Lvm1* on chr13 (Figure S3 in Supplementary Material); the gray nodes represent all other module genes. The linear regression shows that each module gene correlates with LVM in a fashion that is directly proportional to their connectivity index (defined as the log2 transformation of the connectivity value calculated by WGCNA) (*r*^2^ = 0.49, *p* < 0.0001). The pie chart shows that as much as 14 (out of the total 48) module genes physically originated from chr13. The bar graphs (mean ± SD) show that the connectivity of module genes and their correlation with LVM is proportional to their classification in the three respective groups (**p < 0.01; ***p < 0.001).

A graphic representation of the thistle2 module, where the size of each node/gene and the thickness of each edge is proportional to their connectivity and strength, respectively, is shown in Figure 3. We separated genes in the module into three distinct groups according to a combination of criteria that included their connectivity, the physical position of their locus and/or their genetic linkage with LVM. The first group comprised to a cluster of 11 eQTL genes all comprised within a 8 Mb interval on chr 13 (from positions 60.8–68.7 Mb), among which 10 genes corresponded to the most connected genes in the module. The second group in the module comprised five genes that were not physically located on chr13, but are all trans-eQTL genes whose eQTL profiles matched that of *Lvm1* (Figure S5 in Supplementary Material). The third group comprised all other genes from the module, three of which also belonged to chr13. Altogether, 14 (out of the total 48) genes originated from chr13, this number being much higher than the number of genes expected to originate randomly from chr13: given that the ENTREZ database reports that 808 out of all 20,369 genes originate from chr 13, one expects only 1.9 genes to originate from chr13 in a module of 48 genes. In addition, another five trans-eQTLs also showed linkage to the same locus chr13 (Figure S5 in Supplementary Material). A second important observation was that the correlation coefficient of each gene with LVM was directly proportional to its connectivity value (Figure 3). Both connectivity and correlation decreased progressively across groups in the following order: (1) the physical cluster of 11 genes on chr13; (2) the group of five trans-eQTLs on chr13; and (3) all other network genes.

Although the mQTL of the plum2 module did not match a strong phenotypic QTL for LVM, its genes also correlated with LVM in a manner that was directly proportional to their connectivity. Its general organization was also similar to that of thistle2, as its genes could be divided in three groups as a function of their connectivity (Figure S6 in Supplementary Material). The most connected genes corresponded to a cluster of 22 eQTL genes all contained within a 6 Mb interval on chr 17 (from positions 21–26.5 Mb). These 22 genes were all comprised within the group of the 31 most connected genes in the module.

### Comparisons of co-expression modules

We further tested whether other modules had properties similar to that of thistle2 and plum2. Out of the 49 modules detected by WGCNA, 27 had a clear genetic component, since they showed linkage to one main mQTL [with, for 5 of them, at least one additional mQTL that had a lower LOD score (Table S2 in Supplementary Material)]. Out of the 27 “genetic” modules, 21 had their mQTL on the same chromosome that contributed more genes to the module, with the latter genes clustering within an interval averaging 18.3 ± 10.3 Mb (Table 1). That value was significantly smaller than that of the interval containing genes from the predominant chromosome in the six other modules (51.5 ± 12.1) (Table 1). Since this suggested that genes in the above 21 modules originated from a restricted domain rather than from the entire chromosome, we defined these modules as being “CDD.” We further compared the properties of CDD and non-CDD genetic modules to those of the other 22 “non-genetic” modules (Table 1) (Tables S2 and S3 in Supplementary Material). Although the two types of genetic modules contained a higher proportion of genes that could be defined as cis-eQTLs, the abundance of cis-eQTLs was higher in CDD than in non-CDD modules (Tables S2 and S3 in Supplementary Material). Both types of genetic modules showed linkage to one main mQTL, but corresponding LOD scores were higher in CDD than in non-CDD genetic modules. In all modules, we calculated the relative levels of connectivity of genes from the predominant chromosome by dividing their mean connectivity by that of module genes originating from other chromosomes, and found that that value was higher in CDD modules than in non-genetic modules (Table 1). Although non-CDD genetic modules also contained (compared to non-genetic modules) a higher proportion of genes from one predominant chromosome, these genes did not originate from restricted domains, nor did they show increased levels of relative connectivity (Table 1).

**Table 1:** **Properties of different types of modules**.

Characteristics	CDD genetic modules (a)	Genetic non-CDD modules (b)	Non-genetic modules (c)	ANOVA/*post hoc* tests
Mean distance between genes from pred. chrom. (Mb)	18.3 ± 10.3	51.5 ± 12.2	45.21 ± 17	*P* = 4.2e−08
				*P*^ab^ = 1.6e−05
				*P*^ac^ = 2.75e−07
				*P*^bc^ = 5.97e−01
Percentage of cis-eQTL genes	24.3 ± 10	6.09 ± 3.96	2.7 ± 1.7	*P* = 3.76e−13
				*P*^ab^ = 1.84e−06
				*P*^ac^ < 2e−16
				*P*^bc^ = 5.3e−01
Mean LOD of main mQTL	11.5 ± 3.3	4.3 ± 0.7	~	*P* = 2.24e−09
Percentage of genes from predominant chromosome	41.3 ± 11.5	12.3 ± 2.08	11 ± 2	*P* < 2e−16
				*P*^ab^ = 5.93e−10
				*P*^ac^ < 2e−16
				*P*^bc^ = 9.1e−01
Relative connectivity of genes from predominant chromosome (ratios)	3.2 ± 0.9	1 ± 0.1	0.95 ± 0.13	*P* = 5.08e−15
				*P*^ab^ = 4.17e−09
				*P*^ac^ < 2e−16
				*P*^bc^ = 9.91e−01

*CDD, chromosome domain-driven. All values are mean ± SD. The last column lists the *P*-values for the ANOVA tests, followed by those of the *post hoc* tests (when more than three groups are tested; groups compared are represented by letters in superscript)*.

By analyzing gene expression in different tissues from several mouse RIS, we have previously reported on the existence of chromosome domains that contain genes that could all be linked as cis-eQTLs to one same locus within these domains (which we called “cis-eQTL clusters”) (18). On the basis of data from hearts of AxB/BxA RIS mice, these domains corresponded to regions averaging 221.9 ± 130 kb and contained in average a cluster of 4.23 ± 1.9 highly co-expressed cis-eQTL genes. We compared the locations of the cis-eQTL clusters to that of the peaks of the CDD modules mQTLs (Table S4 in Supplementary Material). In 14 of 21 CDD modules, the peak of the mQTL coincided very closely with the locus of the previously reported cis-eQTL clusters. Five of these CDD modules also contained genes from additional cis-eQTL clusters located on the same chromosome, but at some further distance from the mQTL peak (Table S4 in Supplementary Material). In the thistle2 module, 6 out of the 11 most connected genes corresponded to one of the cis-eQTL clusters we identified in our previous work (18). This cluster contained six contiguous genes within a 250 kb interval on chr13 that we identified on the basis of eQTL analysis (see above). In the plum2 module, among the 22 most connected genes located within a 6 Mb interval on chromosome 17, 5 of them corresponded to a cluster of 5 neighboring genes located within a 375 Kb interval on chromosome 17, as reported previously (18).

### Structural variants in chromosome domains

**Figure 4:**
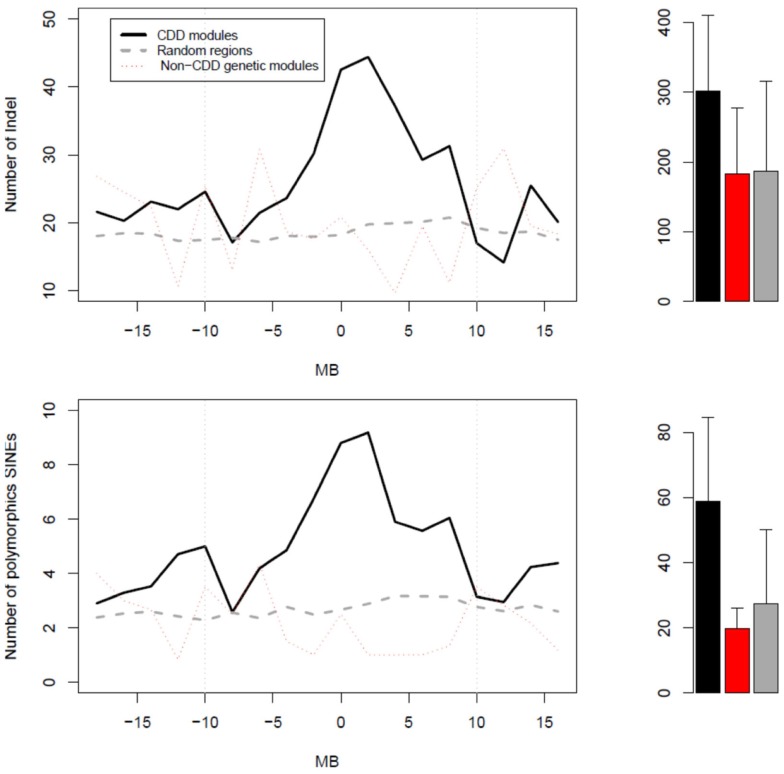
**Profiles of abundance of structural variants (top graph) or polymorphic SINEs (bottom graph) in three types of regions**. The zero Mb position corresponds: (1) for CDD modules (black) and non-CDD genetic modules (red), to the peak of their mQTL; and (2) for random regions (gray), to random SNPs. On right side, the bar graphs represent the mean values of abundance of either structural variants or polymorphic SINEs in regions of 20 Mb centered on the “zero Mb” position. For structural variants, the *p*-value for the ANOVA test was 0.000327 (*post hoc* black vs. gray: *p* < 0.0001). For polymorphic SINEs, the *p*-value for the ANOVA test was 4.41e−09 (*post hoc* black vs. red: *P* = 5.7e−04; *post hoc* black vs. gray:*P* = 2.9e−09).

Given that a great number of genes in CDD modules appeared to originate from restricted chromosome regions, we tested whether the latter had particular physical properties. We thus examined the abundance of either indels or polymorphic SINEs in regions surrounding the mQTL peaks of CDD modules (Figure 4). In CDD modules, both indels and polymorphic SINEs were significantly more abundant in regions of 10 Mb on both sides of the mQTL peaks than in regions surrounding either the mQTL peak of non-CDD genetic modules or 500 random polymorphic SNPs from across the genome. The abundance of these polymorphic elements was distributed in a progressively decreasing gradient fashion around a maximum that coincided with the mQTL of the CDD module.

## Discussion

Previous studies that explored gene expression as intermediate links between genomic markers and quantitative phenotype have focused mostly on c3-eQTLs linked to single genes. In particular cases where the effects of single allelic variants were highly penetrant, this approach has made it possible to identify allelic gene variants that are causal to cardiovascular quantitative traits, including (among others) cardiac LVM (36, 37), fatty acid and glucose metabolism (38), hypertension (39), and dystrophic cardiac calcifications (40). In the current study, the analysis lead to the identification of not just one, but eight c3-eQTLs that all clustered within the confidence interval of the *Lvm1* QTL. Moreover, co-variant analysis showed that that for all c3-eQTLs, their relation to the LVM phenotype was more causal than reactive. The findings, despite the functional utility, did not make it possible to prioritize any of the eight c3-eQTLs over the others as a possible causal variant.

As an alternative to c3-eQTL analysis, we used WGCNA to detect modules of inter-connected genes. Two out of 49 detected modules correlated with LVM. The one with the best correlation contained all eight c3-eQTLs near *Lvm1*, and was linked itself to a mQTL whose profile matched that of *Lvm1*. Although previous studies have reported on modules linking to a QTL overlapping with the interval of a phenotypic QTL (15, 16), these studies did not explain why a genetic determinant could lead to coordinate changes in the expression of genes in the module. In the present study, 27 gene co-expression modules showed linkage to one main mQTL; among them, 21 modules had their mQTL on the same chromosome that contributed more genes to the module. Since the latter genes clustered within a small genetic interval, we considered that they belonged to “CDD modules.” The two modules correlating with LVM corresponded to such CDD modules. More detailed analysis showed that the most connected genes in the module were in fact the ones originating from the chromosome domain. Within these modules, the functional importance of coordinate regulation was highlighted by the fact that, beyond their individual level of expression, genes in the module correlated with LVM as a function of their inter-connectivity.

In mouse RIS, we have previously reported on the existence of chromosome domains that contain genes that could all be linked as cis-eQTLs to one same locus within these domains, and were thus named “cis-eQTL clusters” (18). These domains: (1) did not correspond to either haplotype blocks nor to regions with different recombination rates; (2) showed enrichment for some (but not all) types of transposable elements; (3) corresponded to regions (as reported in several ENCODE projects) showing enrichment for binding sites to several transcription factors; and (4) contained cis-eQTLs that showed much higher levels of co-expression than control regions with similar gene density and haplotypic structure. Further comparisons of the gene co-expression modules detected by WGCNA with our previous study showed that 14 of 21 of the CDD modules had an mQTL peak that coincided very closely with the locus previously reported to link to cis-eQTL clusters. Of note, the power of detection of cis-eQTLs in the AxA/BxA RIS (and of phenotypic QTLs in our previous study) may be partly limited by the fact that the panel comprised only 24 strains. However, WGCNA represents an approach that is very different from cis-eQTL analysis, and it is striking to observe that both analyses detected many of the same genomic regions. Despite the potential limitation in power of the cis-eQTL analysis, the results thus suggest that the genomic regions detected by the combined analyses correspond to chromosome domains harboring particular features. Moreover, the overlap between one of such chromosome domains with a phenotypic QTL indicates that changes in expression level of genes within one of such domains may associate with quantitative differences in a complex trait.

Only a minority of complex traits are expected to result from situations where the effect of a single allelic variant is so penetrant that it can explain a large portion of the variance of the trait (6, 41). The current findings indicate a mechanism whereby genetic variants within chromosome domains may associate to phenotypic changes via coordinate changes in the expression of several genes. Of note, since recent sequencing studies in humans have shown that membership of genes in co-regulated modules is predicted not only by linear proximity, but also by proximity due to three-dimensional chromosomal configuration (as detected by Hi-C analyses) (42), it is possible the coordinate changes in gene expression result from chromatin modifications induced by the polymorphic structural variants in these domains. However, most (if not all) currently available ENCODE data were obtained using only single mouse strains: one needs to acquire additional data across several strains to fully understand whether chromosome-domains result from polymorphisms affecting binding of transcription factors and/or remodeling of chromatin.

Beyond the mechanisms leading to coordinate changes in the expression of genes within domains, one remaining challenge is to understand how such changes may lead to quantitative differences in phenotypic complex traits. Although polymorphisms may affect expression of several genes within chromosome domains, it remains possible that only one of the genes whose expression is affected is causally linked to the phenotype. However, currently available information about genes with altered expression within the chr13 domain is not sufficient to incriminate one of them as causative to increased LVM. As summarized in Table S5 in Supplementary Material, six out of eight of them are poorly annotated and have little known role or function, and only two of them (*Ctsl* and *Cdk20*) have been reported in the literature for their potential role in the heart. In our panel of RIS, mice carrying the A/J allele at the chr13 locus have higher LVM than their counterparts carrying the C57BL/6 allele. Although *Ctsl* has been reported as having anti-hypertrophic activities in genetically modified mice (43, 44), this effect is contrary to that observed in our RIS mice, as its expression is increased in mice that carry the A/J allele and display higher LVM. In contrast, increased expression of Cdk20 has been reported to promote hypertrophy in cardiomyocytes (45); however, its expression in hearts from in mice that carry the A/J allele is only 13% higher than in their counterparts carrying the C57BL/6 allele. Thus, a full understanding of how genes in the domain on chr13 may regulate LVM will require additional functional studies on the properties of each gene. Alternatively, linkage of the locus to the phenotype may implicate more than just one gene. In support of this possibility, recent studies using zinc-finger nuclease mutagenesis of six consecutive genes have shown that multiple genes co-segregating at a single locus in a hypertension GWAS may all have properties compatible with a blood pressure regulating role (46). Regardless of whether the causative mechanism implicates one or more genes, our findings suggest that candidate genes to consider for complex traits should not be restricted to just those genes in closest proximity to associated SNPs or linkage peaks, but should include inter-connected genes as well.

## Conflict of Interest Statement

The authors declare that the research was conducted in the absence of any commercial or financial relationships that could be construed as a potential conflict of interest.

## Supplementary Material

The Supplementary Material for this article can be found online at http://www.frontiersin.org/Journal/10.3389/fcvm.2014.00011/abstract

## Footnotes

^1^http://www.genenetwork.org

^2^http://www.sanger.ac.uk/cgi-bin/modelorgs/mousegenomes/snps.pl
