# Supplementary material for: Dual Linkage of a Locus to Left Ventricular Mass and a Cardiac Gene Co-Expression Network Driven by a Chromosome Domain
Source: Front Cardiovasc Med. 2014 Dec 10;1:11. doi: 10.3389/fcvm.2014.00011 (PMC4668859; doi:10.3389/fcvm.2014.00011)

SUPPLEMENTAL MATERIAL

Dual linkage of a locus to left ventricular mass and a cardiac gene co-expression network driven by a chromosome domain

Supplemental Table S1: Characteristics of 8 c3-QTL genes on chromosome 13

gene name	start position	gene exp LOD	correlation with LVM	drop Lvm1 LOD	residual Lvm1 LOD
Golm1	59.73	5.9	0.73	3.59	1.01
4930486l24rik	60.94	7.4	0.58	2.21	2.39
Habp4	64.26	5.9	0.70	3.47	1.13
Cdk20/ccrk	64.53	10.8	-0.68	3.11	1.49
Cdc14b	64.29	7.7	0.68	3.00	1.60
Ctsl	64.46	13.6	0.66	2.94	1.66
Zfp367	64.24	8.9	-0.63	2.67	1.93
Aaed1	64.39	14.5	0.60	2.34	2.26

The start positions refer to the transcription start sites of corresponding genes on chromosome 13 (in Mb). The “drop Lvm1 LOD” corresponds to the decrease in value of the LOD socre of Lvm1 after using the expression level of each corresponding gene as a covariate for QTL mapping. The “residual Lvm1 LOD” corresponds to the LOD score of Lvm1 obtained after using the expression level of each corresponding gene as a covariate for QTL mapping.

Supplemental Table S2: Characteristics of module QTLs (mQTLs) of "genetic" modules

Type of module	Module Names	main mQTL position	mQTL LOD	matching cis-eQTL clusters
CDD	bisque4	15@76.4	12.64	15@76.6
CDD	brown4	9@44.5	11.67	9@44.2 / 9@44.8
CDD	darkolivegreen	11@98.8	10.15	11@96.8
CDD	darkred	6@144.9	6.43	6@145.1
CDD	darkturquoise	9@105.9	17.9	9@106
CDD	lightcyan1	13@113.2	9.9	13@113.7
CDD	midnightblue	4@133.4	11.7	4@113.9
CDD	orangered4	17@33.1	16.76	17@34
CDD	palevioletred3	4@154.5	9.32	4@155.2
CDD	plum1	2@26.1	6.83	2@25.2
CDD	royalblue	11@57	7.35	11@58
CDD	skyblue3	1@172.9	16	1@172.9
CDD	thistle2	13@64.8	12.2	13@64.3
CDD	cyan	2@113.4	14.1	2@103.9
CDD	floralwhite	7@105.8	11.8	~
CDD	ivory	12@110.3	11.7	~
CDD	lightsteelblue1	13@47	6.8	~
CDD	mediumpurple3	7@50.6	9.3	~
CDD	plum2	17@9.08	16	~
CDD	sienna3	14@46.2	13.53	~
CDD	thistle1	5@119.3	10.35	~
non-CDD	darkslateblue	1@94.8	4.13	
non-CDD	orange	1@116.9	3.9	
non-CDD	pink	17@84.1	3.85	
non-CDD	saddlebrown	17@89.2	4.1	
non-CDD	salmon	12 @101.7	5.8	
non-CDD	skyblue	16@20.4	4.16	

The modules showing linkage to one main mQTL were divided into those showing evidence (or not) of being "chromosome-domain driven"(CDD). The names of each module correspond to those given by the WGCNA program. The characteristics of each mQTL correspond to the number of the chromosome harboring the mQTL, followed by its position (in Mb) on the chromosome. For 16/21 CDD modules, the peak of their mQTL was in very close vicinity of the position of a "cis-eQTL cluster", as reported by us previously (doi:10.1534/g3.112.005488).

Supplemental Table S3: Properties of genes from “genetic“ modules

Type of module	Module Names	% of cis-eQTLs among module genes	% of module genes from predom. chrom.	Predom. Chrom. #	mean Interval between genes from pred. chr. (Mb)	relative connectivity of genes from predom. chrom. vs. others
CDD	bisque4	22.41%	50.00%	15	8.13	2.56
CDD	brown4	42.37%	54.24%	9	11.02	2.87
CDD	darkolivegreen	32.38%	56.19%	11	13.34	2.38
CDD	darkred	15.17%	27.59%	6	16.13	2.14
CDD	darkturquoise	24.65%	42.25%	9	16.50	3.53
CDD	lightcyan1	14.08%	22.54%	13	14.25	4.34
CDD	midnightblue	40.12%	30.23%	8	22.58	1.57
CDD	orangered4	16.46%	58.23%	17	12.67	3.94
CDD	palevioletred3	27.91%	34.88%	4	11.89	2.95
CDD	plum1	26.25%	51.25%	2	50.13	3.58
CDD	royalblue	17.01%	26.53%	11	19.21	2.53
CDD	skyblue3	31.87%	41.76%	1	19.26	5.18
CDD	thistle2	32.65%	30.61%	13	13.13	3.23
CDD	cyan	15.61%	39.31%	2	38.05	3.25
CDD	floralwhite	22.39%	41.79%	7	21.66	3.95
CDD	ivory	10.29%	32.35%	12	5.55	2.81
CDD	lightsteelblue1	36.11%	36.11%	13	18.03	2.59
CDD	mediumpurple3	29.11%	56.96%	7	29.18	2.96
CDD	plum2	27.27%	56.36%	17	19.31	2.68
CDD	sienna3	3.13%	30.21%	14	8.61	5.17
CDD	thistle1	22.92%	47.92%	5	15.99	2.11
**mean**		**24.29%**	**41.30%**		**18.3**	**3.2**
**SD**		**9.97%**	**11.48%**		**10.3**	**0.9**
genetic non-CDD	darkslateblue	3.57%	10.71%	8	58.56	0.88
genetic non-CDD	orange	2.82%	10.56%	9	41.67	1.09
genetic non-CDD	pink	2.95%	10.82%	5	55.89	0.88
genetic non-CDD	saddlebrown	10.26%	14.53%	11	34.92	1.09
genetic non-CDD	salmon	5.06%	11.80%	4	68.43	1.00
genetic non-CDD	skyblue	11.86%	15.25%	9	49.27	0.76
**mean**		**6.09%**	**12.28%**		**51.5**	**1.0**
**SD**		**3.97%**	**2.08%**		**12.1**	**0.1**

Supplemental Table S4: Properties of genes from "non-genetic" modules

Type of module	Module Names	% of cis-eQTLs among module genes	% of module genes from predom. chrom.	Predom. Chrom. #	mean Interval between genes from pred. chr. (Mb)	relative connectivity of genes from predom. chrom. vs. others
non-genetic	black	2.29%	11.11%	11	32.14	1.04
non-genetic	blue	1.60%	8.68%	11	41.88	0.77
non-genetic	brown	4.09%	8.64%	2	52.58	1.15
non-genetic	darkgreen	6.29%	9.79%	2	62.61	1.15
non-genetic	darkgrey	4.93%	14.08%	10	54.85	1.04
non-genetic	darkmagenta	3.49%	8.33%	11	35.08	0.86
non-genetic	darkorange	3.67%	9.29%	10	43.45	0.91
non-genetic	darkorange2	0.00%	8.96%	14	24.44	0.85
non-genetic	green	3.37%	10.67%	7	62.93	1.01
non-genetic	greenyellow	2.67%	10.67%	3	44.76	0.99
non-genetic	grey60	1.86%	9.32%	9	35.42	1.12
non-genetic	lightcyan	1.54%	12.62%	11	40.86	0.90
non-genetic	lightyellow	6.49%	14.29%	2	62.59	0.81
non-genetic	paleturquoise	0.00%	11.32%	5	57.00	0.91
non-genetic	salmon4	2.17%	13.04%	11	19.50	0.89
non-genetic	steelblue	3.48%	13.04%	5	49.19	0.80
non-genetic	tan	1.64%	14.21%	10	30.12	0.96
non-genetic	turquoise	3.82%	8.79%	9	45.22	0.73
non-genetic	violet	0.94%	10.38%	5	58.83	0.81
non-genetic	white	2.48%	8.26%	19	6.27	1.02
non-genetic	yellow	2.42%	10.48%	3	52.77	1.13
non-genetic	yellowgreen	1.09%	11.96%	1	82.19	1.04
**mean**		**2.74%**	**10.81%**		**45.2**	**1.0**
**SD**		**1.74%**	**2.01%**		**17.0**	**0.1**

Supplemental Table S5: : Additional information concerning the eight c3-QTL genes on chromosome 13

gene symbol	Gene name	A vs B allele cardiac expression	Potential function in heart	Gene ontology annotation
Golm1	golgi membrane protein 1	↑ 32%	None reported	nucleus organization
4930486l24rik	Testin-2	↑ 19%	None reported	cysteine-type peptidase activity
Habp4	hyaluronic acid binding protein 4	↓ 63%	None reported	hyaluronic acid binding
Cdk20/ccrk	cyclin-dependent kinase 20	↑ 13%	Promotes LVH	cyclin-dependent protein kinase activity
Cdc14b	cell division cycle 14B	↑ 35%	None reported	phosphoprotein phosphatase activity / DNA repair
Ctsl	cathepsin L	↑ 51%	Protects against LVH	cysteine-type peptidase activity
Zfp367	zinc finger protein 367	↑ 54%	None reported	regulation of transcription, DNA-templated
Aaed1	AhpC/TSA antioxidant enzyme domain containing 1	↑ 18%	None reported	Not determined

For *Cdk20* and *Ctsl*, information concerning their potential roles in the heart is as indicated in the discussion of the manuscript.

LEGENDS OF SUPPLEMENTAL FIGURES

Supplemental Figure S1: QTL mapping profiles for LVM and 9 cis-eQTL genes from chr13 whose expression correlates significantly with LVM. **Fig. S1a**: genome-wide profiles; **Fig. 1b**: profiles at the level of chr13. For LVM, the horizontal black and grey lines correspond to the threshold levels of significant and suggestive QTLs, respectively. The light grey vertical lines represent the confidence interval for the phenotypic QTL *Lvm1*. The darker vertical lines correspond to the confidence intervals for the QTL of each respective gene. The confidence intervals of the first 8 cis-eQTLs fell within the boundaries of the confidence interval for *Lvm1*; the confidence interval of the eQTL for Fastkd3 fell just outside of these boundaries.

Supplemental Figure S2: QTL mapping profiles of the 8 c3-QTL genes on chr 13, either before (black lines) or after (gray lines) using LVM as a covariate for QTL mapping. In each case, residual variance is still such that each cis-eQTL peak remains clearly detectable.

Supplemental Figure S3: Effects of varying parameters on the prediction strength of the network (in comparison to the network constructed with the default parameters). Effects are shown for varying: 1) the soft thresholding β variable (*upper left); 2) the deepSplit variable of the cutreeDynamic function (*upper right); 3) the minClusterSize of the cutreeDynamic function (*lower left*); and 4) the cutHeight values of the mergeCloseModules function (*lower righ*). The vertical lines correspond to the prediction strength found for the network with default values (corresponding to a value = 1). Prediction strength was maintained to high values over a wide range of variations of the last three values.

Supplemental Figure S4: Effects of variations of the soft thresholding β variable on the membership of most connected gene within detected modules. The black and grey lines corresponded to the top 20% and 40% most connected genes, respectively.

Supplemental Figure S5: QTL mapping profiles (at the level of chr13) for LVM, for the thistle2 module, and 5 trans-eQTLs. The peak of the mQTL and that of all 5 trans-eQTLs fell within the boundaries of the confidence interval for *Lvm1*.

Supplemental Figure S6: Diagram representation and properties of the plum2 the co-expression module. The size of each node is proportional to the connectivity of each corresponding gene; the width of each edge is proportional to the strength of correlation between the two corresponding genes. Each node is color-coded in the following fashion: the red nodes comprised a physical cluster of 22 eQTL genes all contained within a 6 Mb interval on chr 17 (from positions 21 to 26.5 Mb); the pink nodes represent other genes on chr17; the grey nodes represent all other module genes. The linear regression shows that each module gene correlates with LVM in a fashion that is directly proportional to their connectivity index (defined as the log2 transformation of the connectivity value calculated by WGCNA) ($r^2 = 0.37$, $P < 0.0001$). The pie chart shows that as much as 34 (out of a total of 51) module genes physically originated from chr 17. The bar graphs (mean ± SD) show that the connectivity of module genes and their correlation with LVM is proportional to their classification in the three respective groups (*$P < 0.05$; **$P < 0.01$; ***$P < 0.001$).

Supplemental Fig. S1a

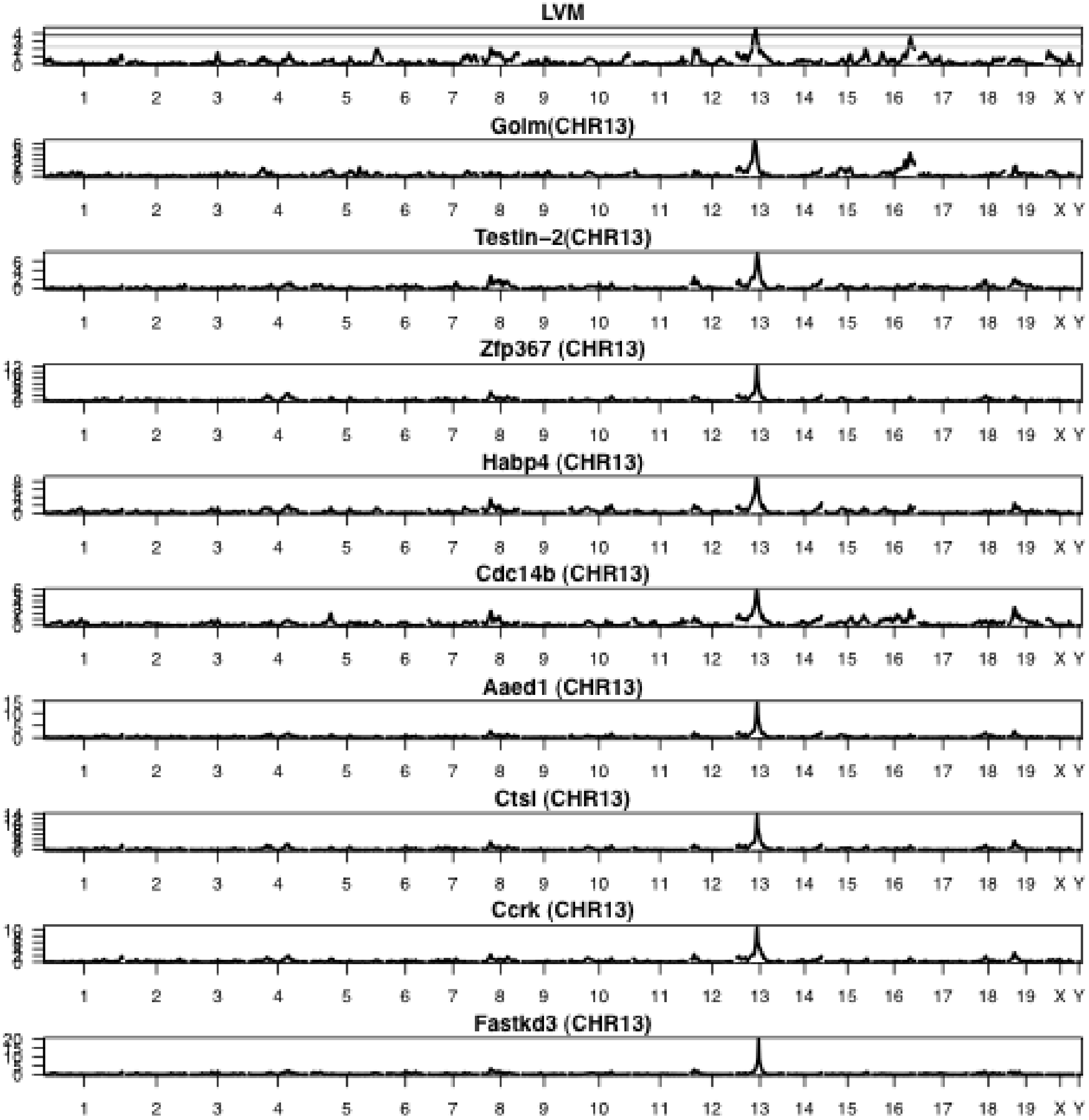

Supplemental Fig. S1b

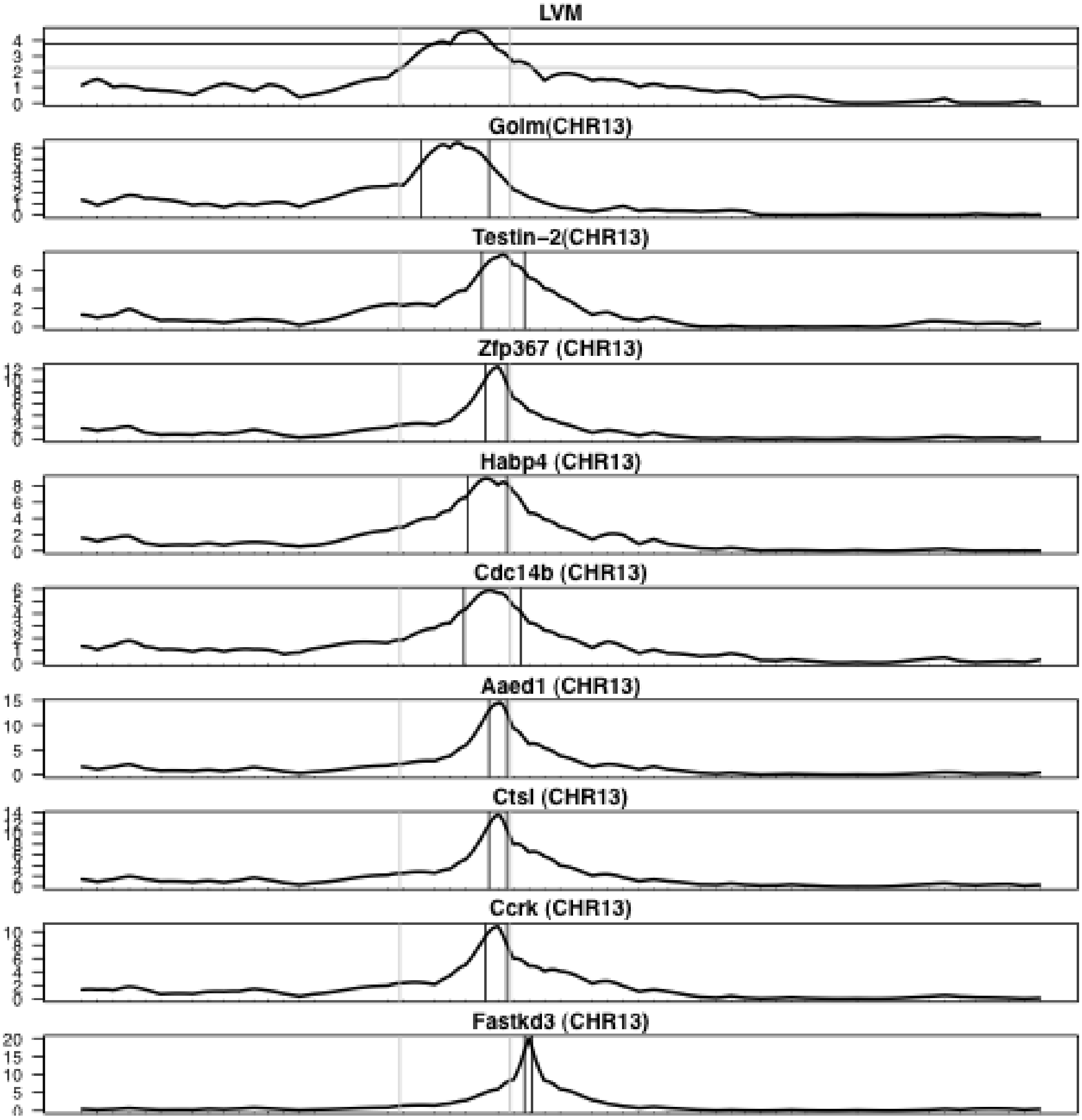

Supplemental Fig. S2

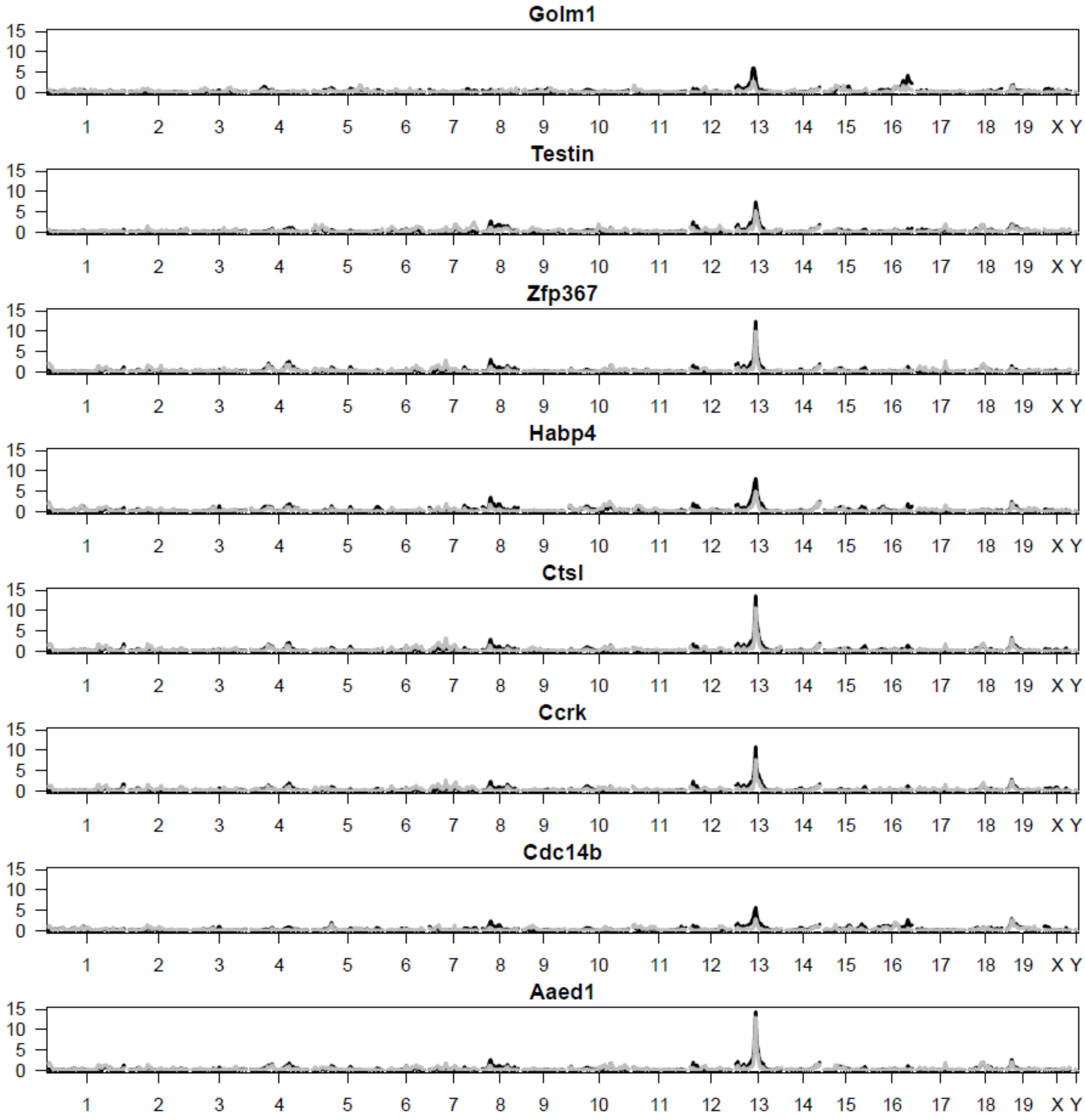

Supplemental Fig. S3

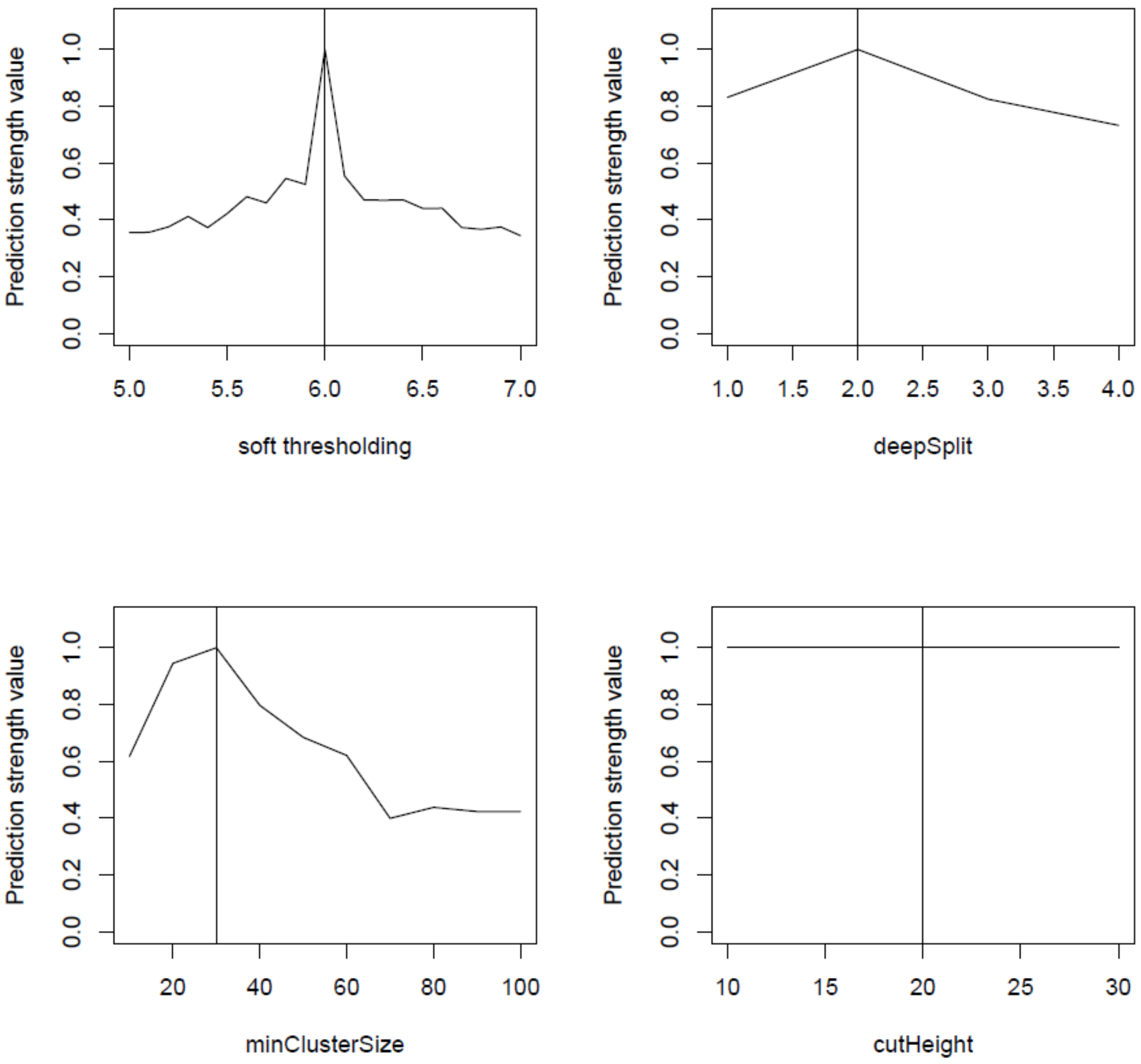

Supplemental Fig. S4

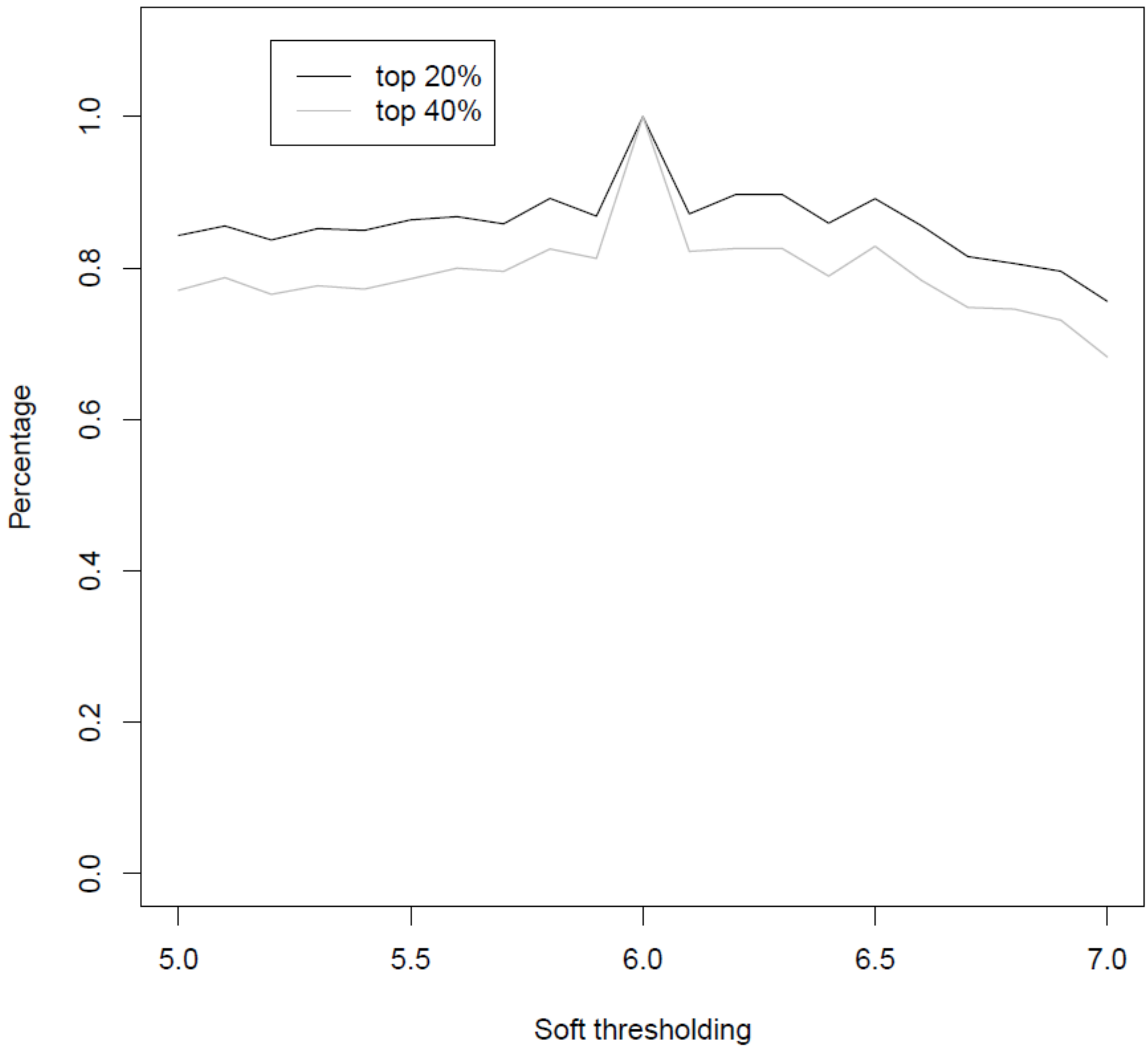

Supplemental Fig. S5

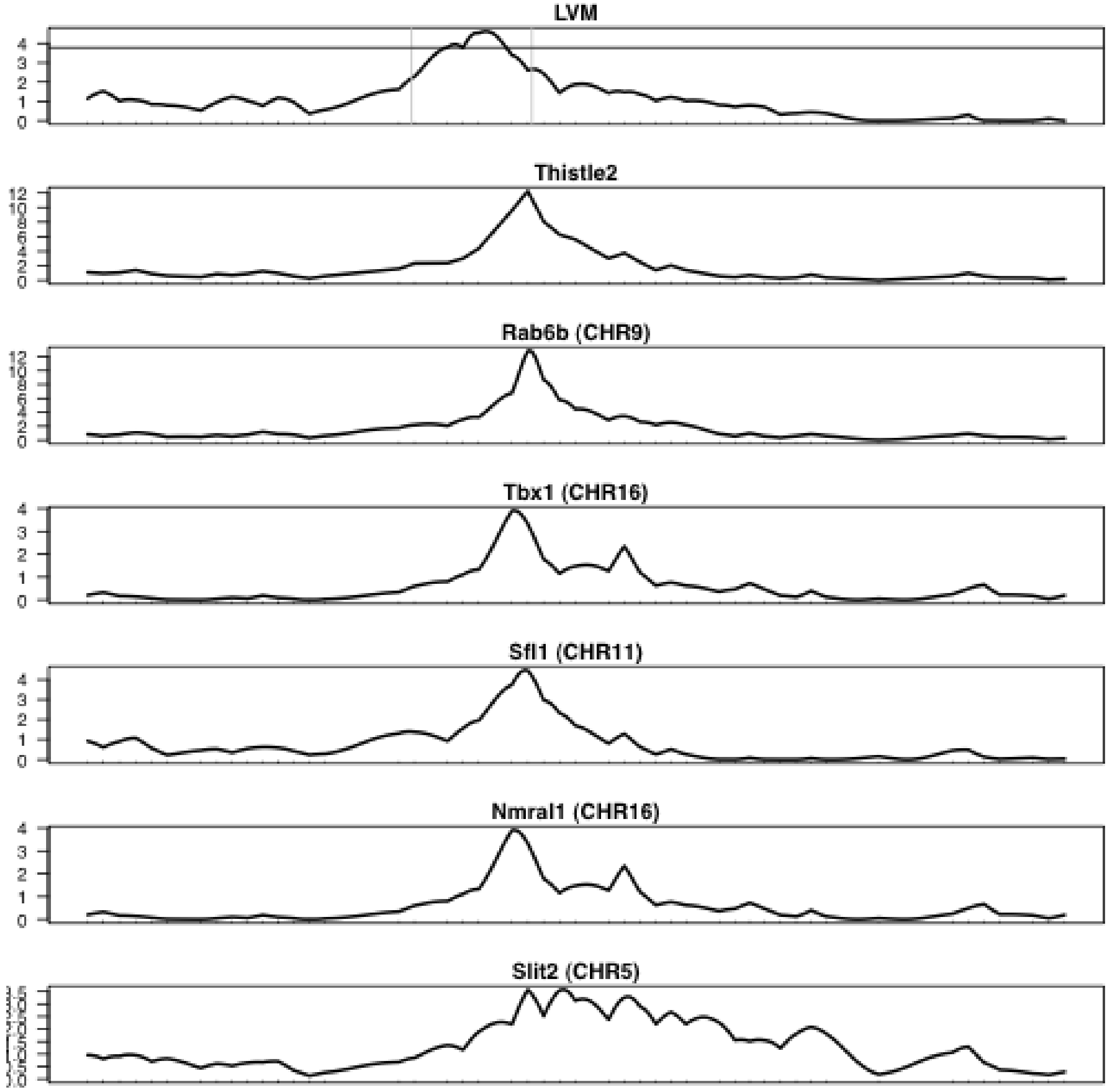

Supplemental Fig. S6

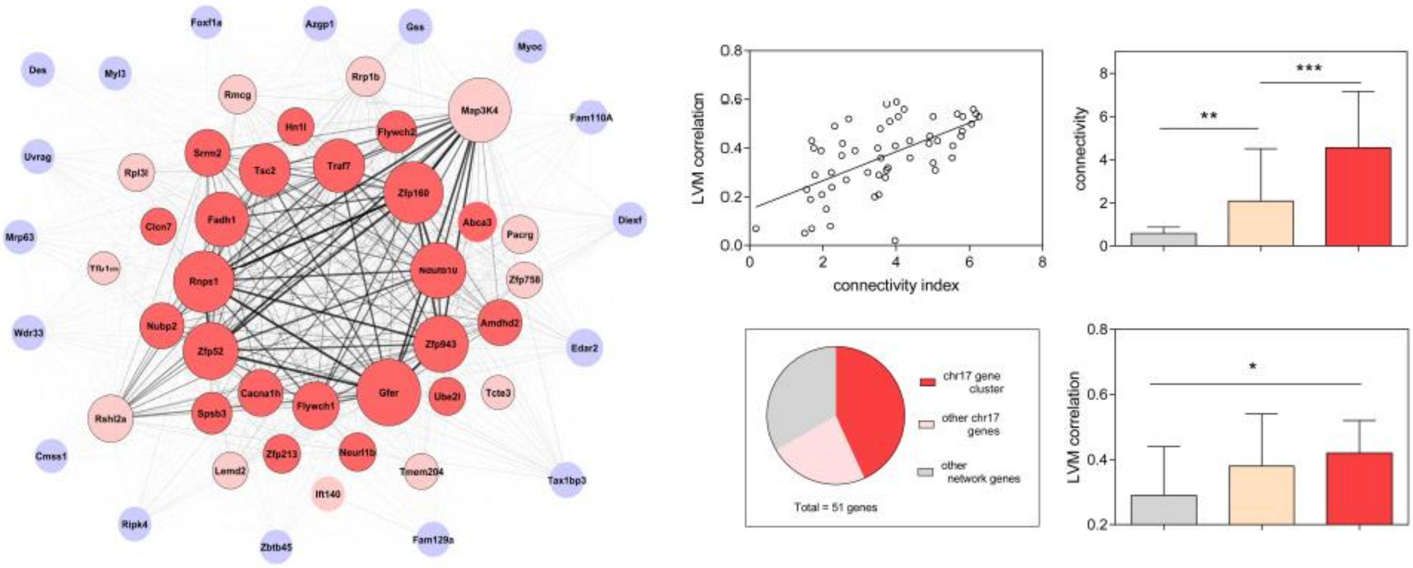

Supplement: Supplementary file 1 [file Presentation_1.PDF]
